# Ceremonial Ayahuasca in Amazonian Retreats—Mental Health and Epigenetic Outcomes From a Six-Month Naturalistic Study

**DOI:** 10.3389/fpsyt.2021.687615

**Published:** 2021-06-09

**Authors:** Simon G. D. Ruffell, Nige Netzband, WaiFung Tsang, Merlin Davies, Antonio Inserra, Matthew Butler, James J. H. Rucker, Luís Fernando Tófoli, Emma Louise Dempster, Allan H. Young, Celia J. A. Morgan

**Affiliations:** ^1^Department of Psychological Medicine, Institute of Psychiatry, Psychology and Neuroscience, King's College London & South London and Maudsley NHS Foundation Trust, Bethlem Royal Hospital, Beckenham, United Kingdom; ^2^College of Life and Environmental Sciences, Washington Singer Laboratories, University of Exeter, Exeter, United Kingdom; ^3^Department of Health and Applied Sciences, University of the West of England, Bristol, United Kingdom; ^4^Kings College London, Institute of Psychiatry, Psychology and Neuroscience, London, United Kingdom; ^5^Neurobiological Psychiatry Unit, Department of Psychiatry, McGill University, Montreal, QC, Canada; ^6^Interdisciplinary Cooperation for Ayahuasca Research and Outreach (ICARO), University of Campinas, São Paulo, Brazil

**Keywords:** ayahuasca, epigenetic, psychedelic, mental health, trauma, ceremony, retreat, DMT

## Abstract

Ayahuasca is a natural psychoactive brew, used in traditional ceremonies in the Amazon basin. Recent research has indicated that ayahuasca is pharmacologically safe and its use may be positively associated with improvements in psychiatric symptoms. The mechanistic effects of ayahuasca are yet to be fully established. In this prospective naturalistic study, 63 self-selected participants took part in ayahuasca ceremonies at a retreat centre in the Peruvian Amazon. Participants undertook the Beck Depression Inventory (BDI-II), State-Trait Anxiety Inventory (STAI), Self-compassion Scale (SCS), Clinical Outcomes in Routine Evaluation-Outcome Measure (CORE-OM), as well as secondary measures, pre- and post-retreat and at 6-months. Participants also provided saliva samples for pre/post epigenetic analysis. Overall, a statistically significant decrease in BDI-II (13.9 vs. 6.1, *p* < 0.001), STAI (44.4 vs. 34.3 *p* < 0.001) scores, and CORE-OM scores were observed (37.3 vs. 22.3 *p* < 0.001) at post-retreat, as well as a concurrent increase in SCS (3.1 vs. 3.6, *p* < 0.001). Psychometric improvements were sustained, and on some measures values further decreased at 6-month follow-up, suggesting a potential for lasting therapeutic effects. Changes in memory valence were linked to the observed psychometric improvements. Epigenetic findings were equivocal, but indicated that further research in candidate genes, such as sigma non-opioid intracellular receptor 1 (SIGMAR1), is warranted. This data adds to the literature supporting ayahuasca's possible positive impact on mental health when conducted in a ceremonial context. Further investigation into clinical samples, as well as greater analyses into the mechanistic action of ayahuasca is advised.

## Introduction

Ayahuasca, meaning “vine of the soul” in the Quechua language (1), is a natural psychoactive plant brew traditionally used for medicinal and spiritual purposes by indigenous populations throughout the Western Amazon basin (2). Scientists first became aware of the Amazonian use of ayahuasca around 150 years ago (3), although its use likely far predates this (4), with some evidence suggesting it may have been used for over 1,000 years (5). In the 1930s, it became introduced to religious settings in small Brazilian urban centres; by the 1980s, prevalence of use had spread to larger cities internationally, with syncretic churches integrating ayahuasca as part of their communions. The most famous of these being the Santo Daime and the União do Vegetal (6). Today, Westerners travel to South America in increasing numbers to participate in ayahuasca rituals, primarily seeking improved insight, personal growth, and emotional or physical healing (7).

The ayahuasca brew is usually prepared by boiling the broken stems of the *Banisteriopsis caapi* vine, alongside leaves from the *Psychotria viridis* shrub or leaves of the *Diplopterys cabrerana* (8). *B. caapi* is rich in the β-carboline alkaloids harmine, harmaline, tetrahydroharmine, amongst others, which act as potent reversible monoamine oxidase inhibitors (MAOIs) (9). Pharmaceutical form MAOIs are widely used as antidepressants (10). *P. viridis* and *D. cabrerana* also contain the psychedelic tryptamine N,N-dimethyltryptamine (DMT), an agonist of the serotonin 2A receptor (5HT_2A_R) and sigma non-opioid intracellular receptor 1 (SIGMAR1). Agonism of these receptors have been associated with antidepressive and anxiolytic effects (11, 12).

When consumed together, the MAOIs in *B. caapi* renders the DMT from *P. viridis* orally active by preventing its deamination (which usually occurs *via* monoamine oxidase in the gastrointestinal tract). This allows for its uptake into the central nervous system, inducing powerful psychedelic effects which often last for ~4–6 h (13). While MAOIs and DMT are both somewhat psychoactive independently, unique phenomenology and pharmacodynamics arise from their interaction in ayahuasca brews (9, 14, 15). A recent review of the literature suggests that such effects are likely synergistic as opposed to simply additive (16).

The ayahuasca experience has been characterised by profound alterations to one's sense of self and reality, emotional and cognitive processing, and spatiotemporal orientation (17). Visual phenomena are also often reported, ranging from colourful geometric patterns to vivid dream-like experiences, alongside transient dissociation, enhanced introspection and initial anxiety followed by euthymia (9, 15, 18–20). Furthermore, ayahuasca's effects have been linked to transcendental and mystical experiences, such as being connected to spirit realms in traditional Amerindian perspectives, and divinity in religious contexts (21). Researchers attempt to measure these experiences using the Mystical Experience Questionnaire (22).

Ayahuasca is safe when used with due caution (23) and has a low dose tolerance and addictive potential (24). Rapid and sustained antidepressant and anxiolytic effects have been shown in both animals (25, 26), and humans (27–30). More broadly, ayahuasca has been associated with improved psychosocial well-being, quality of life, and positive traits such as assertiveness, confidence, optimism, and emotional maturity as well as decreases in neuroticism (25, 31–33). Subacute “after-effects” of ayahuasca include increased mindfulness, ability to decentre and reduced self-judgment and inner reactivity (34). Qualitative reports of greater self-love and compassion have also been suggested, however quantitative measurements have yet to be adequately established (35).

Ayahuasca experiences have been likened to that of intense psychotherapy (36), with a number of authors suggesting it as a treatment candidate for trauma and related disorders (37–39), potentially *via* reprocessing of autobiographic and emotional memories which can be elicited with the ayahuasca-induced dream-like state (17). Anecdotally, abuse victims and recovered addicts report that through ayahuasca-induced visions, they were able to retrieve long-forgotten traumatic memories to work through, which served as a basis for personal life restructuring (40). Neurological evidence also suggests that ayahuasca increases activity in the left hemisphere amygdala and parahippocampal gyrus, areas associated with memory and emotional arousal (41). Despite growing speculation for ayahuasca's efficacy as an intervention for trauma-related conditions, supporting evidence in these conditions is primarily anecdotal, and we suggest further studies.

It is possible that ayahuasca has direct pharmacological effects on trauma-related neurobiology. It has been proposed that the alkaloids present in ayahuasca act *via* the sigma non-opioid intracellular receptor 1 (SIGMAR1) to promote neurogenesis, synaptic plasticity, memory reconsolidation, and fear extinction (42). SIGMAR1 is a stress-responsive neuro-receptor found primarily on the surface of the endoplasmic reticulum. Rodent models of stress-induced downstream SIGMAR1 receptor activation have indicated its potential as a target in post-traumatic stress disorder [PTSD (43)]. Other candidate receptors include FKBP5, which is strongly associated with stress response pathways, and primarily as a co-chaperone of the glucocorticoid receptor activity; it has been implicated in the pathogenesis of stress-related disorders (44). Changes in the DNA methylation pattern within *FKBP5* has also been suggested as a potential proxy marker for response to meditation treatment in PTSD (45).

Thus far, most empirical human studies on ayahuasca have been carried out amongst Brazilian syncretic church members (46), with a small number of studies investigating the use of the brew in retreat centres following a traditional framework (33, 47, 48) and neoshamanic settings (49, 50). While it is important for researchers to investigate ayahuasca use in its varied contexts and traditions, this has been limited by the brew's illicit status in most countries.

In this study, we attempted to ascertain whether ceremonial ayahuasca use may be associated with positive effects on mental health when used in an indigenous framework with foundations in the Shipibo traditions of the Peruvian Amazon. We set out to investigate levels of depression, anxiety, self-compassion and global distress in healthy volunteers before and after an Ayahuasca retreat.

We included secondary measures of autobiographical memory and childhood trauma to investigate potential mediating effects. Measures of subjective mystical experiences were also recorded (33, 51). Lastly, we looked to explore mechanistic hypotheses for potential benefits of ayahuasca. Epigenetic changes *via* DNA methylation of three candidate genes with stress-induced psychopathology were explored; namely FKBP5, BDNF, and SIGMAR1 (44, 52, 53).

We hypothesise that psychological outcomes will improve immediately following the ayahuasca retreat and be maintained at 6-month follow up. Furthermore, these changes will correlate with higher scores on the Mystical Experience Questionnaire post-retreat, and Childhood Trauma Questionnaire scores taken pre-retreat. Finally, changes in DNA methylation of candidate genes may be observed post-retreat vs. pre-retreat.

## Methods

### Participants and Design

The study was conducted at the Ayahuasca Foundation (AF), an ayahuasca retreat and research centre, located in the Amazon rainforest near Iquitos, Peru. The sample group were self-selected.

This was an observational, naturalistic study. Individuals who signed up to the retreat were electronically informed about the research by experimenters the fortnight prior to their retreats commenced, and again upon arrival in Iquitos before transferring to the retreat site. Prospective participants were provided an information sheet and the option to ask questions before giving informed consent to participate in the study before each retreat (the research team were not involved in the recruitment to the retreats, nor dosing of participants). Participation was voluntary with rights to withdraw at any time.

Prior to acceptance onto the retreats, each participant was required to complete an online screening questionnaire on the centre's website. The questionnaire requires information on mental, physical health conditions, and any medications taken. Exclusion criteria includes those with a known diagnosis of psychosis, schizophrenia, bipolar affective, and personality disorders from attendance. All inclusion criteria were determined by the collaborative organisation, the AF.

This study has been approved by the institutional research ethics committee (#CLESPsy000893 v2.0) and complies with the declaration of Helsinki. The research team included a doctor with recognised primary medical qualifications who was present for the duration of the retreats to provide medical assistance if necessary.

### Preparation for Retreat—“Washout Period”

For 2 weeks' prior to attending the retreat, each participant was given instructions by AF to engage in a “washout period,” abstaining from any substances (prescribed and non-prescribed) with possible or known interactions with the constituents of ayahuasca. Furthermore, in order to reduce serum tyramine levels and minimise potential side effects, dietary restrictions on red meats, salt, sugar, and fats were also advised by AF. Lower tyramine levels lessen the likelihood of headaches, nausea, and increased cardiovascular activity which can result from the brew's MAOIs (54).

### Procedure

Ayahuasca was administered to participants in a traditional Shipibo setting adapted for tourists. Retreats varied in length between 8 days to 1 month, including 8-day (four ayahuasca ceremonies), 2-week (six ayahuasca ceremonies), 3-week (nine ayahuasca ceremonies), and 1-month (11 ayahuasca ceremonies) retreats. It was not compulsory for individuals to participate in all ceremonies offered, therefore the researchers recorded the number of ceremonies that each individual participated in.

Ayahuasca ceremonies generally commenced around 20:00, lasted ~5 h, and were led by the local curandero (shaman) with assistance from four to five specially trained facilitators employed by AF. The ceremony space (i.e., the “maloka”) is a round wooden building where single mattresses for each participant are laid out in a circle along the perimeter. A bucket each for “purging” was also provided, due to the brew's typical emetic effect. Participants were instructed to prepare their mindset and set “intentions” regarding what they hoped to achieve leading up to retreats and before ceremonies. Participants were advised to not have physical or verbal contact with one another for the duration of the ceremonies. Ceremonies were undertaken in darkness, with the curandero and facilitators singing traditional medicine songs (i.e., “icaros”) throughout, and providing appropriate care when necessary (e.g., supporting participants to the bathroom). On average, participants consumed ~150 ml of the prepared ayahuasca brew, presented by the curandero at the beginning of the ceremony.

Standardised questionnaires were administered to participants prior to their first ceremony (pre-), the day after their last ceremony (post-), and 6 months after their final ceremony. The pre-retreat data was completed by participants on laptops in a quiet space in the hotel the night before travelling into the jungle for their first ceremony; 4 ml of saliva was also collected under the guidance of researchers at this time point for epigenetic analysis. Post retreat measures were completed on laptops in a quiet space at the retreat site on the morning before travelling back into Iquitos, 4 ml of saliva was again collected. The 6-month follow up questionnaires were collected electronically *via* email. Qualitative data was also collected throughout the retreat and is included a separate article.

### Measures

#### Beck Depression Inventory—Second Edition

The 21 item BDI-II is one of the most widely used psychometric tests for measuring depression severity. It is composed of items relating to depression symptomatology such as hopelessness, irritability, cognitions such as guilt or feelings of being punished, as well as fatigue, weight loss, and lack of sexual interest (55). For the purposes of this study, we used the following cut-off points as recommended by the authors of the BDI-II: “not depressed” (0–13), “mild depression” (14–19), “moderate depression” (20–28), “severe depression” (29–63) (56).

#### State-Trait Anxiety Inventory

The STAI is a 40-item psychological inventory measuring two types of anxiety—State Anxiety, or anxiety about an event, and Trait Anxiety, or anxiety as a personality characteristic. Higher scores indicate higher levels of anxiety (57). In this study for analysis of long-term change, the STAI Trait (STAI-T) score was used rather than the State (i.e., lasting changes moreover how participants felt at the time).

#### Self-Compassion Scale

The 26 item SCS is a validated measure of self-compassion. Alongside a total score, it is comprised of six subscales, including three positive constructs of Self-Kindness, Common Humanity, Mindfulness, and their negative opposite constructs of Self-Judgement, Isolation, and Over-Identification (58).

#### Clinical Outcomes in Routine Evaluation-Outcome Measure

The CORE-OM consists of 34 items measuring Global Distress, which subdivide into four subscales outlining four dimensions comprising Global Distress. These include subjective Well-being, Problems/Symptoms, Functioning, and Risk. The CORE-OM is a widely used initial screening and monitoring clinical tool with high internal and test-retest reliability (59).

#### Childhood Trauma Questionnaire

The CTQ is a 28-item measure inquiring about five types of maltreatment in childhood. These include Emotional Abuse, Physical Abuse, Sexual Abuse, Emotional Neglect, and Physical Neglect, with a three-question screening for false-negative reports of trauma. The CTQ can be used for both clinical and non-clinical samples with strong psychometric properties (60). The CTQ was completed at pre-retreat only.

#### Mystical Experience Questionnaire

The 30 item MEQ is a validated measure of psychedelic-occasioned spiritual/peak experiences. The total score is comprised of four dimensions; Mystical Experience, Positive Mood, Transcendence of Time/Space, and Ineffability (22). The MEQ was administered at post-retreat only to capture participants' perceptions of their ayahuasca experiences. The inventory was scored in relation to the entire retreat, rather than individual sessions.

#### Sentence Completion for Events From the Past Test

The SCEPT is a sentence completion task devised as a sensitive measure of over-generality in autobiographical memory. Participants were required to complete 11 sentence stems in reference to past events (e.g., “When I think back to/of…”). Raters coded for memory specificity and into positive and negative memories.

##### SCEPT Inter-rater Reliability

A coding cheque was conducted on 25% of the data from the SCEPT by two researchers to ensure inter-rater reliability. Intraclass correlation coefficients revealed high levels of agreement between raters across the measure (see Table 1).

**Table 1:** Inter-rater reliability statistics for the SCEPT by intraclass correlation coefficients.

	**Inter-rater reliability**
Total specific memories	0.83
Positive specific memories	0.76
Negative specific memories	0.90
Total general memories	0.85
Positive general memories	0.76
Negative general memories	0.85

### Data Analysis

Data is analysed using SPSS 26.0 (61) and Rstudio Desktop 1.4. In any case where a value was missing from the dataset, the participant was excluded from that particular analysis.

Unless otherwise stated, continuous data is presented as mean (standard deviation; SD). Repeated measures ANOVA with a Greenhouse-Geisser correction were used to compare mean differences across time. *Post-hoc* tests using the Bonferroni corrected pairwise comparisons were then performed to assess significance between time points. Pearson bivariate correlation analysis was used to produce r values.

The paired *t-*test statistic was used to determine if there was a change in DNA methylation at the two candidate genes investigated after ayahuasca administration.

#### Epigenetic Analysis

Saliva samples were collected pre- and post- retreat (2× 4 ml). In total, 55 paired samples (pre- and post- retreat) were obtained. DNA extraction was carried out using Isohelix GeneFiX Saliva-Prep DNA Kit (1 ml protocol) as per manufacturers specifications.

#### Bisulfite Conversion

DNA samples were all diluted to 25 ng/ml using ultra high-quality H_2_O. Bisulfite conversion was carried out using the EZ Methylation—Gold Kit D5005 & 5006 according to manufacturer's instructions. In total, 48 of the 55 paired samples were used for this report's data due to time constraints and plate sizes.

#### Bisulfite Pyrosequencing

The SIGMAR1 assay was designed to span 5 CpGs located in the promoter of the gene, while the FKBP5 assay was a re-designed assay based on one from (62) (see Table 2). Optimisation of these assays was then carried out using fully methylated DNA (positive control) and a negative control. PCR was run for 40 cycles to ensure adequate PCR product for all primers (Stage 1: 95°C for 15 min, Stage 2: 95°C for 15 s, 56°C for 30 s, 72°C for 30 s, Stage 3: 72°C for 10 min). PCR optimisation and bisulfite pyrosequencing included 100% fully methylated positive control.

**Table 2:** Bisulfite pyrosequencing assays.

**Assay**	**Forward primer**	**Reverse primer**	**Sequencing primer**	**Target region coordinates** **(hg19)**
FKBP5	GTTGGGATAATAATTTGGAGTTATAGTG	/5Biosg/CTACCAAATAACTCCTTAAA AAAATAAAAT	GGAGTTATAGTGTAGGTTTT	chr6:35,558,488-35,558,515
SIGMAR1	GTGTGGGGATAGTGAGATTTAGAAT	/5Biosg/CCACCCTAAAACTCCCAACTT	GGGATAGTGAGATTTAGAATG	Chr9:34638039-34638081

Pyrosequencing used to obtain individual gene DNA methylation data per DNA sample, using the Qiagen Pyromark Q48 Autoprep Pyrosequencer. The process was carried out as per manufacturer's instructions. In total, 48 sample discs were loaded with between 10 and 17 μl PCR product and 3 μl magnetic beads. The variation in PCR product was dependent on gene-specific optimisation assays run on the pyrosequencer; if the nucleotide signal peaks obtained from sequencing output had low detection from 10 μl PCR product, more PCR product for the specific assay was added to increase sensitivity for the data collection experiments (FKBP5b = 14 μl, SIGMAR1 = 10 μl). Sequencing primer was 4x diluted in annealing buffer before addition to machine cartridge.

## Results

### Sample Demographics

The sample consisted of 63 participants in total, 35 males (55.6%) and 25 females (44.4%), aged between 19 and 63 (Mean = 37.0, SD = 9.7). Most participants were White (79.4%). In total, 26 were in full-time employment (41.3%), 17 freelance (27.0%), eight unemployed (12.7%), six part-time (9.5%), and six (9.5%) were students. Annually, 25 earned between $10 and 50K (39.7%), 22 between $50 and 100K (34.9%), and the remainder earning either more (14.3%) or less (9.5%).

Forty-eight participants (76.2%) reported no diagnosed physical health problems; three reported hypertension (3.2%), one reported irritable bowel syndrome, one reported seizures (1.6%), and 11 reported “other” conditions (e.g., ankylosing spondylitis, coeliac disease, scoliosis; 17.5%). In total, 42 participants reported no diagnosed psychiatric disorders (66.7%), 15 reported depression, 15 anxiety (19.0%), five ADHD (two comorbid. 4.8%), and five PTSD (4.8%). In total, 27 participants (42.9%) disclosed having experienced problem substance use, including alcohol, tobacco, or caffeine.

In total, 37 (58.7%) stated no previous ayahuasca use, with the rest reporting previous use ranging 1–80 times (Mean = 5.9, SD = 13.2). Number of participants per retreat length in this study were 18 (28.6%) in the 8-day, 12 (19.0%) in the 14-day, 12 (22.2%) in the 21-day, and 19 (30.2%) in the 28-day retreat.

The mean CTQ score in our sample was 48.3 (SD 17.6) Physical abuse = 8.1 (3.7) [ranked “low”], Sexual abuse = 7.8 (5.7) [ranked low], Emotional neglect = 12.5 (5.3) [ranked low], Physical neglect = 8.5 (3.8) [ranked low], Minimisation = 0.1 (0.4) [ranked “minimal”].

### Outcome Measures

**Figure 1:**
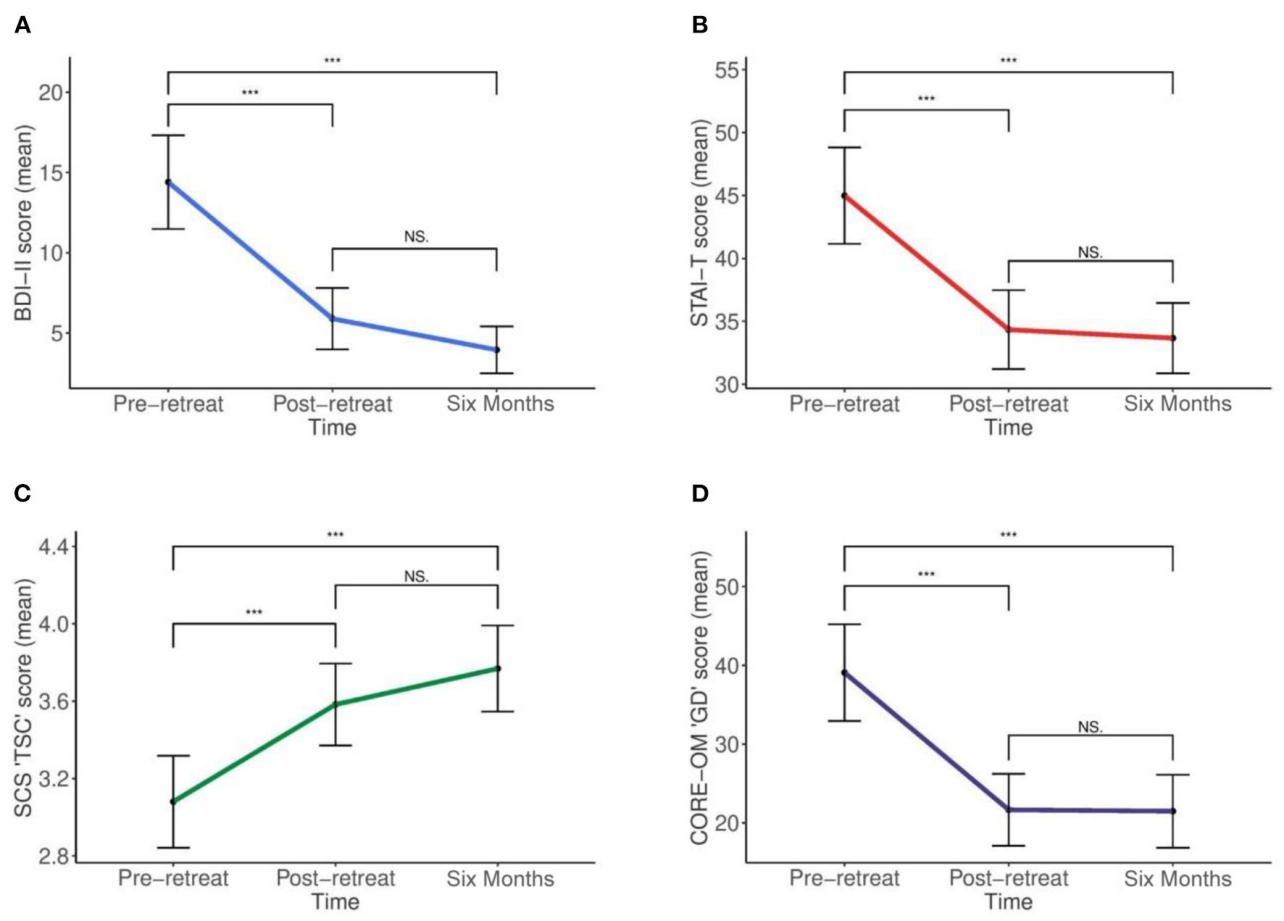
Changes in outcome scores over time. **(A)** Beck Depression Inventory II (BDI-II). **(B)** State and Trait Anxiety Inventory (STAI)—Trait Anxiety Score. **(C)** Self Compassion Scale (SCS)—Changes in Total self-compassion (TSC) score. **(D)** Clinical Outcome Routine in Routine Evaluation (CORE-OM): changes in mean CORE-OM Global Distress (GD) score [NS, non-significant (*P* > 0.05), ***p ≤ 0.001].

Mean outcome scores all differed statistically between time points (see Figure 1, plates A-D) for the BDI-II: *F*_(2, 55)_ = 30.3, *p* < 0.001; STAI-T: *F*_(2, 53)_ = 30.6, *p* < 0.001; SCS: *F*_(2, 53)_ = 21.5, *p* < 0.001; and the CORE-OM: *F*_(2, 55)_ = 21.3, *p* < 0.001. *Post-hoc* tests using the Bonferroni corrected pairwise comparisons revealed a reduction in all severity scores from pre- to post-retreat for the BDI-II; STAI-T; and CORE-OM, which were all statistically significant at the *p* < 0.001 level. Six-month follow-up scores further reduced for the BDI-II; STAI-T; and CORE-OM, which was all statistically significant compared with pre-retreat scores at the *p* < 0.001 level, but not post-retreat scores (BDI-II, *p* = 0.153; STAI-T, *p* = 1.0; CORE-OM, *p* = 1.0), suggesting sustained improvement. For the SCS, there was an increase from pre to post retreat, which was statistically significant (*p* < 0.001); follow-up SCS score further increased and was significant compared with pre-retreat (*p* < 0.001), but not post-retreat (*p* = 0.138), again suggesting sustained improvement. Only total scores from measures were used in the present analysis, for further detail of subscale means and standard deviations, please see Table 3.

**Table 3:** Summary of means and standard deviations for all scales and subscales.

	**Pre-retreat** ***Mean (SD)***	**Post-retreat** ***Mean (SD)***	**T1–T2** **D (*p*)**	**Six month Follow up** ***Mean (SD)***	**T1–T3** **D (p)**
**BDI-II**	14.4 (11.6)	5.9 (7.6)	0.87 (<0.001)	4 (5.5)	1.15 (<0.001)
**STAI**					
- Trait anxiety	45 (15.1)	34.3 (12.3)	0.77 (<0.001)	33.7 (10.6)	0.87 (<0.001)
- State anxiety	40.1 (13.4)	28 (9.2)	1.05 (<0.001)	30.3 (8.8)	0.86 (<0.001)
**SCS**					
- Total	3.1 (1.1)	3.6 (0.8)	0.57 (<0.001)	3.8 (0.9)	0.78 (<0.001)
- Self kindness	3.1 (1.0)	3.7 (0.8)	0.67 (<0.001)	4 (0.8)	0.95 (<0.001)
- Self judgement	3.2 (1.1)	2.6 (1.1)	0.56 (0.001)	2.4 (1)	0.79 (<0.001)
- Common humanity	3.3 (1)	3.8 (0.9)	0.46 (0.008)	3.8 (1)	0.45 (0.005)
- Isolation	3 (1.2)	2.4 (1.1)	0.46 (0.001)	2.3 (1.1)	0.63 (0.001)
- Mindfulness	3.5 (0.9)	3.9 (0.8)	0.46 (<0.001)	4.1 (0.8)	0.72 (<0.001)
- Over-identification	3.1 (1.1)	2.6 (1.1)	0.42 (0.015)	2.5 (1.2)	0.54 (<0.001)
**CORE-OM**					
- Global distress	39.1 (24.4)	21.7 (18.1)	0.81 (<0.001)	21.5 (17.4)	0.83 (<0.001)
- Global distress minus risk	37.5 (22.9)	21.3 (17.8)	0.79 (<0.001)	21.1 (16.7)	0.82 (<0.001)
- Subjective well-being	5.6 (4.4)	2.9 (3.2)	0.69 (0.001)	3.1 (3.2)	0.65 (<0.001)
- Problem/symptoms	18 (10.8)	10.8 (9.5)	0.71 (<0.001)	9.8 (8.7)	0.84 (<0.001)
- Life functioning	14 (9)	7.4 (6.6)	0.83 (<0.001)	8.2 (6.1)	0.75 (<0.001)
- Risk/harm	1.6 (2.8)	0.4 (0.9)	0.59 (0.013)	0.5 (1.2)	0.52 (0.011)
**SCEPT**					
*All Memories*					
- Specific	2.5 (1.9)	2.9 (1.8)		2.3 (1.5)	
- General	8.0 (2.1)	7.8 (1.7)		8.3 (2.0)	
- Omissions	0.5 (1.1)	0.3 (1)		0.5 (1.6)	
*Positive Memories*					
- Total	6.5 (2.2)	7 (2.1)		7.2 (2)	
- Specific	1.7 (1.6)	2.1 (1.6)		1.8 (1.3)	
- General	4.8 (1.9)	4.9 (1.9)		5.5 (2.1)	
*Negative Memories*					
- Total	4.2 (1.9)	3.8 (1.9)		3.3 (1.6)	
- Specific	0.8 (0.9)	0.9 (1.0)		0.5 (0.7)	
- General	3.4 (1.6)	2.9 (1.7)		2.8 (1.4)	
**CTQ**					
- Total	48.3 (17.6)	–		–	
- Physical abuse	8.1 (3.7)	–		–	
- Sexual abuse	7.8 (5.7)	–		–	
- Emotional neglect	12.5 (5.3)	–		–	
- Physical neglect	8.5 (3.8)	–		–	
- Minimisation	0.1 (0.4)	–		–	
**MEQ**					
- Total	–	115.1 (30.1)		–	
- Mystical experience	–	57.6 (17.3)		–	
- Positive mood	–	24.0 (6.2)		–	
- Transcendence	–	22.1 (6.3)		–	
- Ineffability	–	11.8 (3.6)		–	

*Effect sizes (D) for four main outcome measures are reported as Cohen's d. P-values obtained from repeated ANOVAs are corrected for multiple comparisons through Bonferroni post-hoc analysis*.

**Figure 2:**
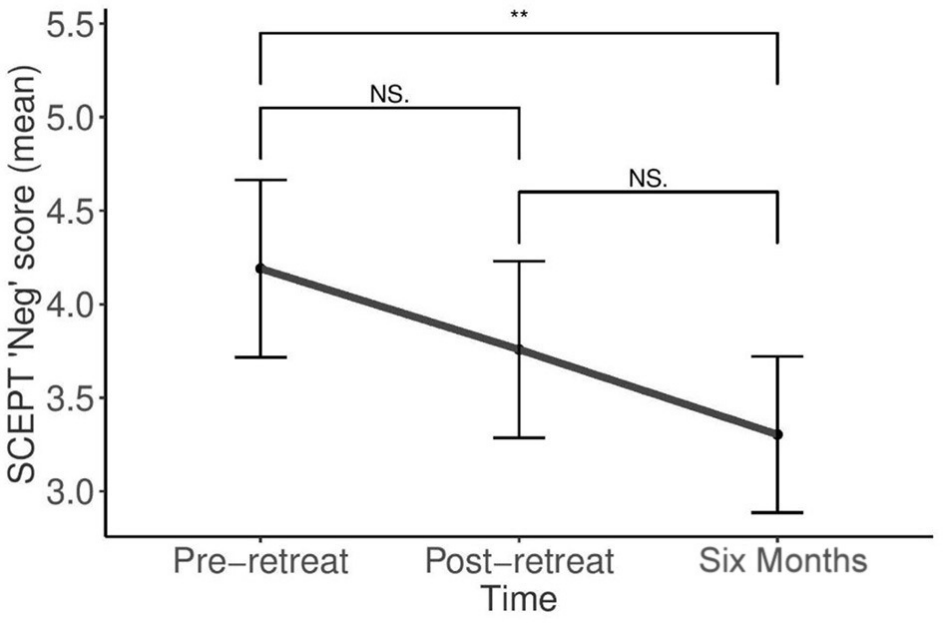
Sentence Completion for Events from the Past Test (SCEPT): Changes in SCEPT negative valanced memory scores over time [NS, non-significant (*P* > 0.05), **p ≤ 0.01].

No significant changes in memory specificity were found on the SCEPT. However, new variables of total positive and negative memory scores for each time point were computed to assess changes in memory valance. Mean SCEPT negative valanced memory scores differed statistically significantly between time points, *F*_(2, 110)_ = 5.68, *p* < 0.005. While not reaching significance, there were trend levels of reduction in negatively valanced memories from pre to post-retreat, and post retreat to follow-up. There were, however, significant reductions in negative valanced memories from pre-retreat to follow-up (*p* = 0.004), suggesting improvement over time (Figure 2).

#### Subsample Meeting Screening Cut-Off for Depression

At pre-retreat, 31 of the participants met cut-offs for depression based on BDI-II scores as detailed under *Methods* (11 mild depression, 11 moderate depression, and nine severe depression). This subsample had a mean score of 24.2 (median 23.0). At post-retreat, 24 (77.4%) were no longer depressed, four had mild depression, one moderate, and two severe (mean 8.7, median 5.0). At 6-month, 24 were remained not depressed, two mild, and one severe (four missing values) (mean 5.2, median 4.0). Changes in BDI-II score in the depressed subsample was significant between time points, *F*_(2, 25)_ = 55.5, *p* < 0.001.

Repeated measures ANOVA found no significant changes in the SCEPT “total specific” and total “overall general” subscales, suggesting no change in memory specificity as a function of time in the depressed subsample. There was also no difference between total CTQ scores in the depressed (*n* = 31, mean 50.5, SD 19.0) and the non-depressed (*n* = 32, mean 46.2, SD 16.2) subsample on *t*-test, *t*(61) = 0.97, *p* = 0.915.

#### Correlation Analysis With Number of Ceremonies, Length of Retreat, and Frequency of Ayahuasca Use Prior Retreat

*Note: As not all participants took part in every ayahuasca ceremony offered on retreat, “number of ceremonies” was recorded as it varied between participants*.

Pearson's correlation was performed between number of ceremonies, length of retreat, and frequency of ayahuasca use prior retreat and improvement scores on the BDI-II, STAI-T, CORE-OM, and SCS. Due to multiple comparisons, alpha was set at 0.01. There were no significant correlations.

#### Predictors of Change in Psychopathology

To minimise the risk of type 1 errors, Pearson's correlations were conducted with CTQ and MEQ total scores and subscales and BDI-II change scores (the latter chosen as a proxy for all outcomes given similar patterns of findings across all outcome measures). Greater change in BDI-II post-retreat was correlated with higher overall CTQ scores (r = 0.318, *p* = 0.011 for overall population and r = 0.393, *p* = 0.029 for clinically depressed population) scores. These figures were however not significantly correlated with BDI-II change at 6-month. In the depressed subsample alone, only the mystical experience subscale of the MEQ was negatively correlated (i.e., those with greater scores had greater improvements in BDI scores) with change in BDI-II post-retreat (r = −0.357, *p* = 0.049). This correlation was not sustained at 6-month and there was no correlation at either time point in the overall sample. For a full breakdown these correlation analyses, please see Table 4.

**Table 4:** Correlation coefficients vs. BDI-II change scores at post-retreat and 6 month follow-up.

	**Pearson correlation coefficient vs. BDI-II post-retreat change score (p)**		**Pearson correlation coefficient (r) vs. BDI-II follow-up change score (p)**	
	**Overall population** **(*n* = 63)**	**Depressed population** **only (*n* = 31)**	**Overall population** **(*n* = 57)**	**Depressed population only** **(*n* = 27)**
**Childhood trauma questionnaire**				
Total score	**−0.318 (0.011)**	**−0.393 (0.029)**	0.189 (0.148)	−0.250 (0.208)
Physical abuse	−0.119 (0.354)	−0.306 (0.095)	0.045 (0.742)	−0.061 (0.764)
Sexual abuse	**−0.263 (0.038)**	−0.345 (0.057)	−0.077 (0.570)	0.071 (0.726)
Emotional neglect	**−0.260 (0.039)**	−0.180 (0.333)	**−0.305 (0.021)**	−0.376 (0.053)
Physical neglect	−0.246 (0.052)	−0.340 (0.061)	−0.195 (0.146)	−0.291 (0.140)
Minimisation/denial	0.105 (0.415)	0.035 (0.854)	0.129 (0.340)	0.151 (0.452)
Emotional abuse	**−0.292 (0.20)**	−0.352 (0.052)	−0.150 (0.266)	−0.286 (0.149)
**Mystical experience questionnaire**				
Total score	−0.061 (0.637)	−0.274 (0.136)	0.067 (0.622)	−0.112 (0.579)
Mystical experience	−0.102 (0.428)	**−0.357 (0.049)**	0.097 (0.475)	0.016 (0.938)
Positive mood	−0.043 (0.737)	−0.351 (0.053)	0.104 (0.441)	−0.194 (0.332)
Transcendence	−0.164 (0.200)	−0.213 (0.250)	−0.116 (0.388)	−0.094 (0.642)
Ineffability	−0.143 (0.265)	−0.338 (0.063)	0.064 (0.636)	−0.101 (0.616)

*Depressed population is those with BDI-II score ≥14 at baselines (four lost to follow-up). Results significant at alpha = 0.05 level are highlighted in bold. Negative correlation equates to greater changes in BDI-II related to higher scores on CTQ and MEQ*.

### DNA Methylation Analysis

BDNF analyses failed due to an error, therefore only SIGMA and FKBP5 were analysed.

#### SIGMAR1

**Figure 3:**
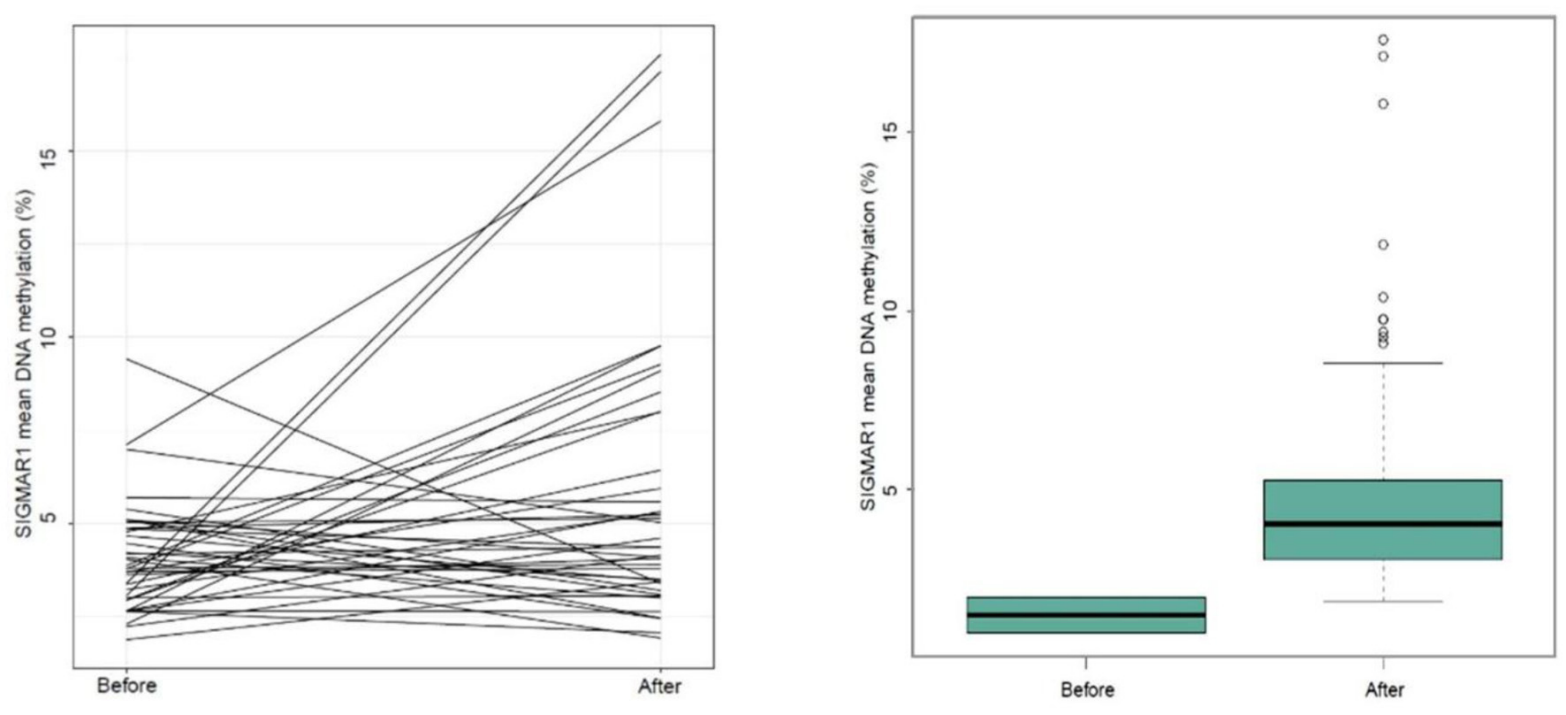
Mean changes in DNA methylation across 5 CPG positions within the SIGMAR1 gene (paired *t*-test *p* = 0.01; *n* = 38).

The SIGMAR1 assay showed a statistically significant increase in DNA methylation across the 5 analysed CpG sites (paired *t*-test: t = 2.58, df = 38, *p* = 0.01) (see Figure 3).

#### FKBP5

FKBP5 DNA methylation did not show any statistically significant change (*p* = 0.13).

#### SIGMAR1 Methylation Correlation Analyses

**Figure 4:**
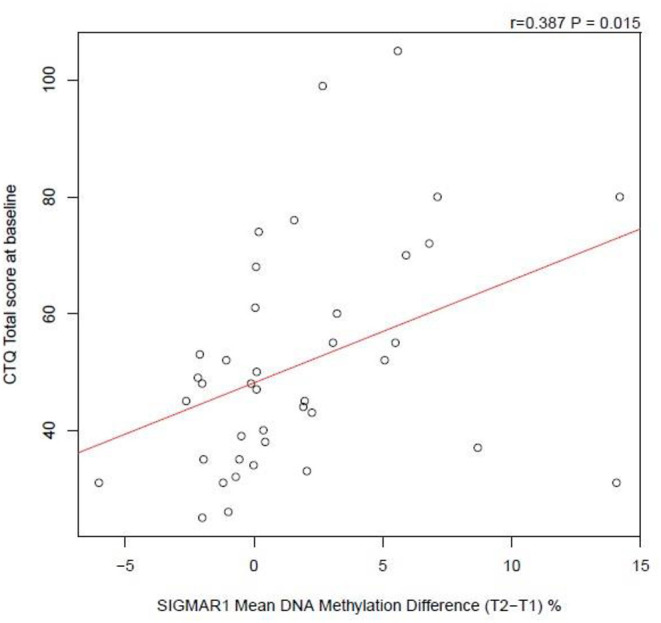
Correlation analysis of CTQ total score at baseline against SIGMAR1 Mean DNA Methylation Difference (from T2 to T1).

Methylation change scores were calculated for SIGMAR1 and Pearson's correlation performed with CTQ total scores. There was a significant correlation (r = 0.387, *p* = 0.015), indicating those with higher childhood trauma had increased methylation changes in SIGMAR1 post retreat (Figure 4). In order to reduce the risk of type I errors, SIGMAR1 methylation changes were correlated with BDI-II as a proxy for all outcome measures; there was no significant correlation in this analysis.

## Discussion

In this naturalistic study we examined the associations between ayahuasca use and a number of mental health outcomes. We found that ayahuasca was associated with reductions in depression, anxiety, and global distress from baseline to post-retreat which were sustained at 6-month follow-up. Many patients meeting clinical scores for depression pre-retreat did not do so after the ayahuasca retreat. We also observed reductions in negative memory valance on a sentence completion task from baseline to after the retreat, with no changes in recall specificity. There was evidence of a change in DNA methylation at loci on the SIGMAR1 receptor gene between pre- and post- retreat.

### Depression

The results of the current study suggest that ayahuasca use in ceremonial settings may be associated with improvements in well-being, particularly depression and its related conditions. It should be noted that although significant improvements in depression symptoms were shown, mean BDI-II ratings in the overall sample pre-retreat were nonetheless below threshold for likely depression diagnoses. To this end, we subsequently conducted further analysis on those within our sample meeting threshold for depression according to the BDI-II at pre-retreat; the majority of those meeting the cut off for depression pre-retreat no longer did so at post-retreat or follow-up. As most within this subsample reported mild depression, it was perhaps unsurprising to find no correlation between change in BD-II score and memory specificity according to the SCEPT on this subsample.

Similarly, we also found no relationship between memory specificity with the BDI-II for our overall sample. This tallies with past research, which has suggested that changes in overgeneral autobiographical memory tends to emerge only when comparing healthy participants to clinical populations with depression (63). Although no changes in recall specificity were found, a reduction in negatively valanced memories from pre-retreat to 6-month follow-up was observed in our total sample. Overgeneralised negative memory is a characteristic in depression (64), and is proposed as an aetiological factor. The observed reduction in the tendency to recall negative information could be a cognitive correlate antecedent of the improvements suggested.

In line with our original hypotheses and data from previous studies (33, 65, 66), we found associations between perceived mystical effects and improved psychometric scores at follow up. Participants in the depressed subpopulation who reported a greater degree of mystical experience improved to a greater degree post-retreat. Within semi-structured interviews conducted with these participants, many reported that the ritualistic element of the ceremonies amplified their perceived mystical states. Analysis of this data was beyond the scope of the current study; and will be presented separately within a qualitative analysis.

Our findings suggesting improvements in depressive outcomes are consistent with previous studies including a small open-label ayahuasca study (27) and a parallel-arm, double-blind randomised placebo-controlled trial with 29 treatment-resistant depression patients assessing ayahuasca vs. placebo (28). Improvements in depressive symptoms are consistent with previous fMRI data, which has revealed attenuated default mode network (DMN) activity following ayahuasca use (67). The DMN is a neural network known to be hyperactive in major depressive disorder patients and those suffering from severe anxiety, underlying ruminations and self-referential processes (68, 69).

### Anxiety and Well-Being

In our study, ayahuasca use was associated with reductions in trait anxiety and improvements in general well-being. These findings are consistent with recent research in traditional frameworks in the Amazon basin (33, 48), neoshamanic settings (49, 50), and church settings previously (70, 71). In one study, ceremonial ayahuasca was found to be associated with reductions in levels of neuroticism, a personality trait underlying anxiety disorders (33). Improvements in CORE-OM scores in our current sample also supports previous evidence suggesting improvements in general well-being and quality of life (33, 48).

### Contextual Factors

Our study did not show direct correlation between the number of ayahuasca ceremonies and outcome measures, ostensibly indicating no potential benefit of engaging in greater numbers of ceremonies. Although individual sessions may be beneficial, the effects resulting from individual psychedelic therapies are often difficult to predict. Qualitative studies suggest transformative experiences may occur spontaneously in individual ceremonies (20). Psychological “breakthroughs” are sometimes even described as independent of the dose and number of sessions (72). That is, improvement in psychological well-being as a result of ayahuasca use may be non-linear. Future research should seek to investigate this further with qualitative measures and by characterising the nature, and not just frequency, of sessions.

The current study took place at a retreat centre that describes itself as “*rooted in the Shipibo ayahuasca tradition*.” Ayahuasca is given at night in a ceremonial setting that takes place in the darkness with minimal contact between participants. Ayahuasca churches usually provide the brew in well-lit rooms to entire congregations who subsequently sing or engage in conversation surrounding topical issues (6). Despite these differences, the results of our study are in line with those based in both syncretic church settings and controlled studies in laboratories. This may suggest that commonalities could be induced by the drug and not just expectancy effects. Similarities that do exist however, may be defining factors in forming outcomes regardless of context. Even laboratory studies in this area of research typically display features seen in ritualistic settings, such as the role of music (28, 73).

The ritualistic context surrounding ayahuasca also appears to play a crucial role in safeguarding and minimising risks associated with its use (74). Without a supportive “*set and setting*” (75), ayahuasca experiences may not produce benefit, and could even be traumatic (74, 76). Clinical data into ayahuasca suggests that a supportive context can also be achieved in laboratory settings (28, 77). It is possible that the likelihood of therapeutic outcomes is dependent on the individual's affect and perceptions within the given context. Outcomes appear to be, at least partly, determined by a perceived sense of safety and/or support whilst entering psychedelic states (78, 79). For some, greatest benefit may be achieved in the Amazon rainforest with a curandero, whilst for others a clinical setting may be more appropriate. Catering for subjective factors which allows the participant to feel optimally safe is therefore imperative.

### Epigenetics

Our results suggest that ayahuasca exposure affects the epigenetic regulation of SIGMAR1. However, the mean increase (2.1% increase) in DNA methylation is small, and it remains unclear if this change in DNA methylation has biological impacts and alterations to gene expression. It is possible that an increase in SIGMAR1 DNA methylation enables increased expression of the receptor, however, this model is less likely, a common DNA methylation rules mean hypermethylation results in transcriptional silencing. At this stage, the implications of these findings are uncertain. However, these findings are the first of their kind and consolidate indications that SIGMAR1 expression is regulated *via* an epigenetic process.

It is possible that the modest changes in methylation in our sample was due in part to the minimal trauma history of many of our participants. Our overall CTQ scores are in line with previous research utilising the CTQ in non-clinical samples (80, 81), and is lower than expected from clinical samples (82)—this is despite our depressed subsample not having a significantly different CTQ score to our non-depressed subsample.

Coupled with previous evidence for the marker's role in trauma (42, 43), the correlation (albeit weak) between childhood trauma and changes in SIGMAR1 methylation, alongside the improvements in mental health outcomes observed in our present sample, we propose that future research should investigate SIGMAR1 as a potential mechanism of action underlying ayahuasca.

## Strengths and Limitations

To our knowledge, this study is the first to investigate the effects of a psychedelic on epigenetics. A fundamental limitation of this study is the absence of a control group and the likelihood of self-selection bias. Given the time and financial sacrifices necessary to take part in ayahuasca ceremonies, it is likely that participants had strong positive a priori expectations.

Participants were subject to more than simply ayahuasca dosing (i.e., contextual factors such as being in a retreat setting, with a group, without internet in the Amazon rainforest), and therefore the placebo effect is likely to be significant. It was also difficult to control for the impact of maturation and life events between post-retreat and 6-month follow up, which further complicates the inference of causality (e.g., nine participants used ayahuasca again during this time and were excluded from the analysis). Additionally, not all participants were naive to ayahuasca prior to their first ceremony at AF, although no correlations were found between frequency of previous use and psychological outcomes in our present sample.

As the researchers had no access to participants' medical records, it was not possible to adequately confirm medical histories of the current sample. It should also be noted that the quantity of ayahuasca given to participants was not standardised. The curandero provided each participant with what they deemed to be an appropriate dose. Although this could be seen as a limitation, given the observational nature of the study it was deemed appropriate to follow the traditional framework rather than intervene in the ceremonies.

### Limitations of Epigenetic Analyses

As biological samples were taken from peripheral cells (i.e., saliva samples), results may not represent epigenetic changes in the central nervous system. The approach taken here is arguably open to bias, as it was a candidate-gene style analysis. Other researchers have suggested the need for neuronal samples to provide valid epigenetic results (83); however, it is unclear how this would be achieved due to ethical and logistical considerations. Our epigenetic analyses were limited to three candidate genes, future studies should continue to assess the potential epigenetic regulation of other genes, including epigenetic changes in genes related to other mental disorders. Other epigenetic mechanisms like microRNAs were not studied in this project, and we suggest future research considers additional methods of analysis. Gene regulation outside of epigenetics, such as alternative splicing as a result of ayahuasca consumption is also a potential area for future research.

## Ayahuasca Tourism

The researchers wish to highlight some issues surrounding “ayahuasca tourism” (84). As interest in ayahuasca continues to grow, so do issues around safety and cultural appropriation. As well as this, lack of regulation throughout Peru, and beyond, has led to individuals labelling themselves as “shamans” without appropriate training and experience (85), potentially resulting in dangerous practises. Furthermore, although ayahuasca is considered a sacrament by various communities, many of the retreat centres in the Amazon are owned by Westerners and the use of ayahuasca and other plants for financial gain has been called into question (6). It is vital that scientists and the public alike proceed cautiously given risks regarding safety and the cultural sensitivity of the practises in question.

## Conclusions

The findings of this study suggest ayahuasca use in a traditional Amazonian setting is associated with significant improvements in a number of mental health outcomes. These changes were sustained at 6-month follow-up without further dosing, suggesting lasting therapeutic potential. Our study is the first study to directly examine epigenetic effects correlated with psychedelic use. These findings support hypotheses that SIGMAR-1 may be involved mechanistically in the positive outcomes of ayahuasca use. Future research should aim to investigate the effects of ayahuasca by increasing the scope of biological markers and exploiting neuroimaging technology in randomised controlled trials of clinical populations.

## Data Availability Statement

The raw data supporting the conclusions of this article will be made available by the authors, without undue reservation.

## Ethics Statement

The studies involving human participants were reviewed and approved by University of Exeter. The patients/participants provided their written informed consent to participate in this study.

## Author Contributions

SR, AI, and CM: conceptualization. SR, NN, WT, and CM: methodology. SR and NN: data collection. WT and MB: data analysis. SR, NN, and WT: writing—original draft preparation. SR, NN, WT, MB, LT, AY, and CM: writing—review and editing. MD and ED: epigenetic analysis. JR, LT, ED, AY, and CM: supervision. All authors contributed to the article and approved the submitted version.

## Conflict of Interest

The authors declare that the research was conducted in the absence of any commercial or financial relationships that could be construed as a potential conflict of interest.
