# Corrigendum: Ceremonial ayahuasca in amazonian retreats—mental health and epigenetic outcomes from a six-month naturalistic study

**DOI:** 10.3389/fpsyt.2023.1304503

**Published:** 2023-10-11

**Authors:** Simon G. D. Ruffell, Nige Netzband, WaiFung Tsang, Merlin Davies, Antonio Inserra, Matthew Butler, James J. H. Rucker, Luís Fernando Tófoli, Emma Louise Dempster, Allan H. Young, Celia J. A. Morgan

**Affiliations:** ^1^Department of Psychological Medicine, Institute of Psychiatry, Psychology and Neuroscience, King's College London & South London and Maudsley NHS Foundation Trust, Bethlem Royal Hospital, Beckenham, United Kingdom; ^2^College of Life and Environmental Sciences, Washington Singer Laboratories, University of Exeter, Exeter, United Kingdom; ^3^Department of Health and Applied Sciences, University of the West of England, Bristol, United Kingdom; ^4^Kings College London, Institute of Psychiatry, Psychology and Neuroscience, London, United Kingdom; ^5^Neurobiological Psychiatry Unit, Department of Psychiatry, McGill University, Montreal, QC, Canada; ^6^Interdisciplinary Cooperation for Ayahuasca Research and Outreach (ICARO), University of Campinas, São Paulo, Brazil

**Keywords:** ayahuasca, epigenetic, psychedelic, mental health, trauma, ceremony, retreat, DMT

In the published article, there was an error in the author list, and author Antonio Inserra was erroneously excluded. The corrected author list, affiliations and author contributions appears below.

Simon G. D. Ruffell^1,2^^*^^†^, Nige Netzband^3^^†^, WaiFung Tsang^4^, Merlin Davies^2^, Antonio Inserra^5^, Matthew Butler^4^, James J. H. Rucker^4^, Luís Fernando Tófoli^6^, Emma Louise Dempster^2^, Allan H. Young^1^ and Celia J. A. Morgan^2^

^1^Department of Psychological Medicine, Institute of Psychiatry, Psychology and Neuroscience, King's College London & South London and Maudsley NHS Foundation Trust, Bethlem Royal Hospital, Beckenham, United Kingdom, ^2^College of Life and Environmental Sciences, Washington Singer Laboratories, University of Exeter, Exeter, United Kingdom, ^3^Department of Health and Applied Sciences, University of the West of England, Bristol, United Kingdom, ^4^Kings College London, Institute of Psychiatry, Psychology and Neuroscience, London, United Kingdom, ^5^Neurobiological Psychiatry Unit, Department of Psychiatry, McGill University, Montreal, QC, Canada, ^6^Interdisciplinary Cooperation for Ayahuasca Research and Outreach (ICARO), University of Campinas, São Paulo, Brazil

## Author contributions

SR, AI, and CM: conceptualization. SR, NN, WT, and CM: methodology. SR and NN: data collection. WT and MB: data analysis. SR, NN, and WT: writing—original draft preparation. SR, NN, WT, MB, LT, AY, and CM: writing—review and editing. MD and ED: epigenetic analysis. JR, LT, ED, AY, and CM: supervision. All authors contributed to the article and approved the submitted version.

The authors apologize for this error and state that this does not change the scientific conclusions of the article in any way. The original article has been updated.

